# Kissing Rectal Ulcers With Associated Occult Rectal Mucosal Prolapse: A Distinct Endoscopic Presentation of Solitary Rectal Ulcer Syndrome in an HIV Positive Patient With Comprehensive Infectious Exclusion

**DOI:** 10.1155/crgm/4644258

**Published:** 2026-09-08

**Authors:** Nakul Ganju, Sneha Rao Adidam, Naga Sai Shravan Turaga, Lakshmi Chirumamilla, Suryanarayana Challa Reddy, Rabia Zafar, Angesom Kibreab

**Affiliations:** ^1^ Department of Internal Medicine, Howard University Hospital, 2041 Georgia Avenue NW, Washington, D.C. 20060, USA, howard.edu; ^2^ AdventHealth Altamonte Springs, 601 E Altamonte Drive, Altamonte Springs 32701, Florida, USA

**Keywords:** HIV, infectious proctitis, kissing ulcer, occult prolapse, rectal bleeding, rectal mucosal prolapse, solitary rectal ulcer syndrome

## Abstract

Solitary rectal ulcer syndrome (SRUS) is a rare benign disorder of the rectum strongly associated with chronic straining and rectal mucosal prolapse, which may be overt or occult. Its endoscopic appearance is highly variable, and atypical presentations can create significant diagnostic challenges, particularly in immunocompromised patients in whom infectious etiologies must be systematically excluded. We report a case of SRUS presenting as simultaneous contralateral kissing rectal ulcers in an HIV positive patient. Histopathology demonstrated the characteristic triad of fibromuscular obliteration of the lamina propria, smooth muscle extension between distorted crypts, and muscularis mucosae hypertrophy, establishing occult internal mucosal prolapse as the underlying mechanism. Comprehensive infectious evaluation including targeted testing for cytomegalovirus, herpes simplex virus, Neisseria gonorrhoeae, and Chlamydia trachomatis including lymphogranuloma venereum serovars was entirely negative. The patient achieved complete symptomatic resolution on conservative therapy with psyllium fiber supplementation at 2‐month follow‐up. This case illustrates an uncommon endoscopic presentation of SRUS, the diagnostic importance of histopathology in the absence of visible mucosal prolapse, and a systematic infectious exclusion approach in an HIV positive patient that may serve as a clinical reference for similar presentations.

## 1. Introduction

Solitary rectal ulcer syndrome (SRUS) is a rare, benign, and frequently underrecognized disorder of defecation characterized by rectal symptoms, variable endoscopic findings, and characteristic histopathologic abnormalities [1–3]. The term solitary is a well‐known misnomer: lesions may be multiple, polypoid, or erythematous, and the anterior rectal wall, while classically affected, is not the only site of involvement. In large series, up to 28% of the patients had multiple lesions and 25% had polypoid or nodular variants [4, 5]. Posterior wall involvement has also been reported [6].

The pathogenesis of SRUS is best understood within the framework of mucosal prolapse syndrome [7]. Chronic straining and rectal intussusception subject the rectal mucosa to repetitive mechanical shear and ischemic injury. Mucosal prolapse in SRUS is frequently occult or internal, not visible at endoscopy, and is inferred from the histopathologic triad of fibromuscular obliteration of the lamina propria, smooth muscle extension between distorted crypts, and muscularis mucosae hypertrophy [2, 3, 8]. Endoscopic absence of overt prolapse does not exclude this mechanism.

In patients with HIV, the differential diagnosis of rectal ulceration is substantially broader. Cytomegalovirus (CMV), herpes simplex virus (HSV), Chlamydia trachomatis including lymphogranuloma venereum (LGV), and Neisseria gonorrhoeae can all produce proctitis and ulcerative rectal lesions that are endoscopically indistinguishable from SRUS [9]. Histopathologic confirmation with targeted infectious testing is therefore essential before attributing rectal ulceration to a benign functional etiology in this population. We report an uncommon endoscopic presentation of SRUS as simultaneous contralateral kissing rectal ulcers in an HIV positive patient, with comprehensive infectious exclusion and complete conservative management outcome.

## 2. Case Presentation

A 64‐year‐old woman with HIV infection, well controlled on antiretroviral therapy with an undetectable viral load, presented with rectal bleeding, tenesmus, and progressively worsening constipation of several years duration. She reported intermittent hematochezia and a persistent sense of incomplete evacuation consistent with obstructed defecation. She denied abdominal pain, diarrhea, fever, or unintentional weight loss. A history of colon polyps was noted. Family history was negative for colorectal cancer and inflammatory bowel disease. She denied digital rectal manipulation and enema use. Physical examination and digital rectal examination were unremarkable, with no palpable mass, induration, or active bleeding. Laboratory studies including complete blood count and comprehensive metabolic panel were within normal limits. Her CD4 count was 800 cells/μL and she had been on antiretroviral therapy for more than 10 years, indicating robust immune reconstitution and making opportunistic or AIDS‐defining etiologies unlikely.

**FIGURE 1:**
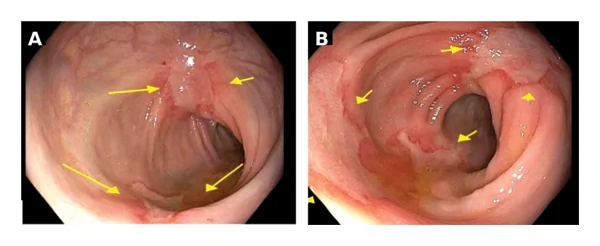
Endoscopic findings at index colonoscopy. (A) Nonbleeding ulcerated mucosa on the anterior rectal wall, 5–7 cm from the anal verge, with well‐demarcated margins and surrounding erythematous, mildly friable mucosa (yellow arrows). (B) Corresponding ulceration on the posterior rectal wall completing the contralateral kissing configuration (yellow arrows). No overt mucosal prolapse was visualized endoscopically.

Colonoscopy revealed two opposing ulcerated mucosal lesions involving the anterior and posterior rectal walls in a kissing configuration, located approximately 5–7 cm from the anal verge (Figures 1A,B). The ulcers were well demarcated with surrounding erythematous, mildly friable mucosa. No overt rectal prolapse was visualized endoscopically. Nonbleeding internal and external hemorrhoids were also identified. The remainder of the colon was normal.

**FIGURE 2:**
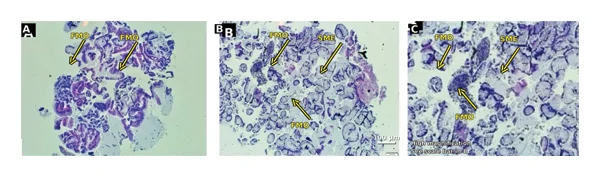
Histopathological findings (hematoxylin and eosin stain). (A) Low magnification view demonstrating markedly distorted glandular architecture with fibromuscular expansion of the lamina propria (FMO, arrows). (B) Higher magnification view showing fibromuscular obliteration of the lamina propria (FMO, arrows) with smooth muscle extension between distorted crypts (SME, arrows). Scale bar = 100 μm. (C) High magnification view of the region indicated in Panel B, more clearly demonstrating fibromuscular obliteration of the lamina propria (FMO, arrows) and smooth muscle extension between crypts (SME, arrows). No viral cytopathic effect, granulomas, dysplasia, or malignancy was identified in any panel. FMO = fibromuscular obliteration; SME = smooth muscle extension.

Biopsies from the ulcer margins were examined by hematoxylin and eosin stain and demonstrated several characteristic features of SRUS (Figures 2A–C). There was marked distortion and architectural disarray of the colonic crypts, with irregular glandular profiles and serpiginous crypt outlines. The lamina propria was replaced by dense fibromuscular tissue, with smooth muscle fibers extending upward between the crypts toward the surface epithelium, the hallmark of fibromuscular obliteration (Figures 2B,C, arrows labeled FMO). Smooth muscle extension between crypts (Figures 2B,C, arrows labeled SME) reflected muscularis mucosae hypertrophy and upward displacement. The surface epithelium showed a fibrinopurulent exudate. There was no evidence of dysplasia, malignancy, granulomas, or viral cytopathic effect. These histopathologic findings are consistent with the established diagnostic criteria for SRUS and reflect the cumulative ischemic and mechanical injury of occult internal mucosal prolapse during chronic straining [2, 3, 7, 8].

Given the patient’s HIV status, comprehensive infectious evaluation was performed. Targeted testing from ulcer tissue and serum was negative for CMV, HSV, Neisseria gonorrhoeae, and Chlamydia trachomatis including LGV serovars, excluding sexually transmitted and opportunistic proctitis. The patient was counseled on the role of chronic straining and obstructed defecation in the pathogenesis of SRUS and was initiated on psyllium fiber supplementation, hydration, and avoidance of excessive straining. At 2‐month follow‐up, she reported complete resolution of rectal bleeding and pain without recurrence of hematochezia. No further endoscopic, topical, or surgical intervention was required.

## 3. Discussion

This case contributes to the clinical literature on SRUS by documenting a simultaneous bilateral kissing ulcer configuration affecting both the anterior and posterior rectal walls concurrently. While single posterior or anterior wall ulcers and multiple nonopposing lesions are recognized SRUS variants [4–6], a comprehensive review of the published literature reveals that the simultaneous contralateral kissing pattern has not been previously characterized as a discrete endoscopic presentation. We, therefore, report this configuration as an uncommon and underrecognized variant of SRUS, while acknowledging that additional case series will be needed to fully define its prevalence and clinical significance. Clinically, contralateral opposing ulcers may more readily raise concern for malignancy or inflammatory bowel disease, making histopathologic confirmation essential before reaching a diagnosis.

The histopathologic findings in this case are instructive. The biopsy demonstrated the complete diagnostic triad of SRUS: fibromuscular obliteration of the lamina propria, smooth muscle extension between distorted crypts, and muscularis mucosae hypertrophy, in addition to crypt architectural disarray and surface fibrinopurulent exudate. Importantly, no overt mucosal prolapse was visible at colonoscopy, illustrating the well‐established principle that rectal mucosal prolapse in SRUS is frequently internal and diagnosed histopathologically rather than endoscopically [7, 8]. These biopsy features, in the absence of viral cytopathic effect, granulomas, or dysplasia, permitted confident exclusion of infectious and neoplastic etiologies.

**FIGURE 3:**
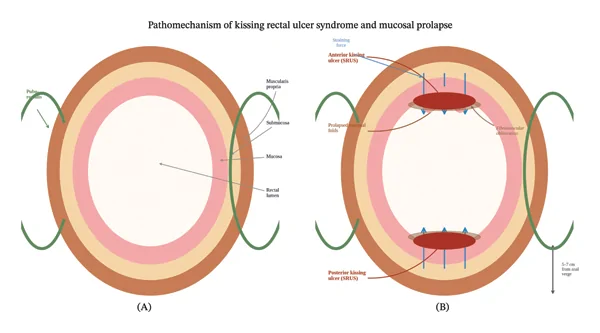
Proposed pathomechanism of the kissing rectal ulcer configuration (schematic hypothesis). (A) Normal rectal anatomy in coronal cross section. (B) During chronic straining, occult internal mucosal prolapse is hypothesized to generate circumferential shear and ischemic forces (blue arrows) applied simultaneously to both the anterior and posterior rectal walls, potentially producing the symmetric opposing fibromuscular obliteration and contralateral kissing ulcer pattern observed at colonoscopy. This mechanistic explanation is consistent with established mucosal prolapse syndrome pathophysiology but cannot be confirmed from a single case report. Ulcer locations at 5–7 cm from the anal verge are indicated.

We propose, as a hypothesis, that the kissing configuration in this case may be explained by the circumferential distribution of shear and ischemic forces generated during internal mucosal prolapse, which could simultaneously injure both the anterior and posterior rectal walls during chronic straining, as illustrated schematically in Figure 3. This mechanistic explanation is consistent with the established pathophysiology of mucosal prolapse syndrome [7] but cannot be confirmed from a single case report and warrants further investigation.

In the setting of HIV, this case demonstrates the importance of a systematic infectious exclusion strategy before attributing rectal ulcers to a benign etiology. Comprehensive testing for CMV, HSV, Neisseria gonorrhoeae, and Chlamydia trachomatis including LGV was negative, and histopathology confirmed SRUS without infectious features. This approach should be considered standard practice when rectal ulcers are encountered in immunocompromised or sexually active patients [9].

Management should begin with conservative measures targeting the defecatory disorder, including fiber supplementation, hydration, and behavioral modification to minimize straining [1, 3, 5]. Biofeedback is effective in patients with pelvic floor dyssynergia [10], while surgical intervention is reserved for refractory cases with overt prolapse [3]. Our patient achieved complete symptomatic resolution with psyllium alone at 2‐month follow‐up.

Several limitations of this case report warrant acknowledgment. First, the follow‐up period of 2 months, while demonstrating complete symptomatic resolution, is relatively short given the known tendency of SRUS to recur, and ongoing longer‐term clinical surveillance is planned for this patient. Second, no defecography or dynamic MRI pelvis was performed to formally document occult rectal mucosal prolapse; the diagnosis rested on histopathologic criteria, which represent the established standard for SRUS diagnosis [2, 3, 7, 8]. Third, the proposed mechanistic explanation for the kissing configuration remains a hypothesis and cannot be confirmed from a single case. Fourth, as this represents a single case report, generalizability is inherently limited, and larger case series will be needed to characterize the full clinical spectrum of this endoscopic presentation.

## Funding

This research received no specific funding from any funding agency in the public, commercial, or not‐for‐profit sectors.

## Disclosure

This study was presented as a poster at the American College of Gastroenterology Annual Scientific Meeting 2024; Sunday Poster Session; Category: Colon; Abstract P0304; Philadelphia, PA; October 2024.

## Consent

Written informed consent was obtained from the patient for publication of this case report and all accompanying clinical images, in accordance with institutional guidelines and the Declaration of Helsinki.

## Conflicts of Interest

The authors declare no conflicts of interest.

## Supporting Information

Additional supporting information can be found online in the Supporting Information section.

## Supporting information


**Supporting Information** CARE checklist: Provided as supporting file.

## Data Availability

The data that support the findings of this study are available from the corresponding author upon reasonable request.
