# Supplementary material for: Kissing Rectal Ulcers With Associated Occult Rectal Mucosal Prolapse: A Distinct Endoscopic Presentation of Solitary Rectal Ulcer Syndrome in an HIV Positive Patient With Comprehensive Infectious Exclusion
Source: Case Rep Gastrointest Med. 2026 Sep 8;2026:4644258. doi: 10.1155/crgm/4644258 (PMC13552358; doi:10.1155/crgm/4644258)
Supplement: Supplementary file 1 [file CRGM-2026-4644258-s001.docx]

**CARE Checklist — Completed for Submission**

*Kissing Rectal Ulcers with Associated Occult Rectal Mucosal Prolapse: A Distinct Endoscopic Presentation of Solitary Rectal Ulcer Syndrome in an HIV Positive Patient with Comprehensive Infectious Exclusion*

Ganju N, Adidam SR, Turaga NSS, Chirumamilla L, Challa Reddy S, Zafar R, Kibreab A. Case Reports in Gastrointestinal Medicine. 2026 (submitted).

| **Topic** | **Item** | **Checklist Item Description** | **Reported on Line / Location** |
| --- | --- | --- | --- |
| **Title** | **1** | The diagnosis or intervention of primary focus followed by the words "case report" | Title page — "Kissing Rectal Ulcers with Associated Occult Rectal Mucosal Prolapse: A Distinct Endoscopic Presentation of Solitary Rectal Ulcer Syndrome in an HIV Positive Patient with Comprehensive Infectious Exclusion" |
| **Key Words** | **2** | 2 to 5 key words that identify diagnoses or interventions in this case report, including "case report" | Title page — solitary rectal ulcer syndrome; kissing ulcer; rectal mucosal prolapse; HIV; infectious proctitis; occult prolapse; rectal bleeding |
| **Abstract** | **3a** | Introduction: What is unique about this case and what does it add to the scientific literature? | Abstract — simultaneous contralateral kissing rectal ulcer configuration as a distinct endoscopic phenotype of SRUS with comprehensive infectious exclusion in HIV |
|  | **3b** | Main symptoms and/or important clinical findings | Abstract — rectal bleeding, tenesmus, worsening constipation; kissing ulcers on opposing rectal walls at colonoscopy |
|  | **3c** | The main diagnoses, therapeutic interventions, and outcomes | Abstract — SRUS confirmed histologically; CMV, HSV, gonorrhea, chlamydia/LGV excluded; conservative therapy with psyllium; complete resolution at two month follow up |
|  | **3d** | Conclusion — What is the main take-away lesson(s) from this case? | Abstract — "This case documents a simultaneous bilateral kissing ulcer configuration as a distinct phenotype of SRUS... and demonstrates a systematic infectious exclusion approach in an HIV positive patient that should serve as a clinical template" |
| **Introduction** | **4** | One or two paragraphs summarizing why this case is unique (may include references) | Introduction — three paragraphs: SRUS endoscopic variability [1–5]; occult mucosal prolapse mechanism [2,3,6,7]; HIV differential diagnosis requiring infectious exclusion [8] |
| **Patient Information** | **5a** | De-identified patient specific information | Case Presentation — 64 year old woman; no name, initials, or identifying information included |
|  | **5b** | Primary concerns and symptoms of the patient | Case Presentation — rectal bleeding, tenesmus, progressively worsening constipation of several years duration, intermittent hematochezia, sense of incomplete evacuation |
|  | **5c** | Medical, family, and psycho-social history including relevant genetic information | Case Presentation — HIV on antiretroviral therapy (undetectable viral load); history of colon polyps; family history negative for colorectal cancer and IBD; denied digital rectal manipulation and enema use |
|  | **5d** | Relevant past interventions with outcomes | Case Presentation — no prior rectal interventions or surgery relevant to this episode |
| **Clinical Findings** | **6** | Describe significant physical examination and important clinical findings | Case Presentation — physical and digital rectal examination unremarkable; no palpable mass, induration, or active bleeding; laboratory studies within normal limits |
| **Timeline** | **7** | Historical and current information from this episode of care organized as a timeline | Case Presentation — (1) Several years: onset of constipation and hematochezia; (2) Referral to gastroenterology for evaluation; (3) Colonoscopy: kissing rectal ulcers identified; (4) Histopathology: SRUS confirmed, infectious testing negative; (5) Conservative therapy initiated; (6) Two month follow up: complete resolution |
| **Diagnostic Assessment** | **8a** | Diagnostic testing (such as PE, laboratory testing, imaging, surveys) | Case Presentation — CBC and metabolic panel (normal); colonoscopy with biopsy; histopathology (H&E); targeted tissue and serum testing for CMV, HSV, Neisseria gonorrhoeae, Chlamydia trachomatis including LGV serovars |
|  | **8b** | Diagnostic challenges (such as access to testing, financial, or cultural) | Discussion — primary diagnostic challenge was broad differential diagnosis in HIV positive patient; infectious etiologies endoscopically indistinguishable from SRUS; kissing configuration potentially suggestive of malignancy or IBD |
|  | **8c** | Diagnosis (including other diagnoses considered) | Case Presentation and Discussion — confirmed diagnosis: SRUS with occult rectal mucosal prolapse; differential diagnoses excluded: CMV proctitis, HSV proctitis, Neisseria gonorrhoeae proctitis, Chlamydia trachomatis/LGV proctitis, inflammatory bowel disease, rectal malignancy |
|  | **8d** | Prognosis (such as staging in oncology) where applicable | Not applicable — SRUS is a benign condition; no staging required. Conservative management expected to be sufficient based on clinical response |
| **Therapeutic Intervention** | **9a** | Types of therapeutic intervention (such as pharmacologic, surgical, preventive, self-care) | Case Presentation — conservative: psyllium fiber supplementation (dietary/pharmacologic); hydration; behavioral modification (avoidance of excessive straining during defecation) |
|  | **9b** | Administration of therapeutic intervention (such as dosage, strength, duration) | Case Presentation — psyllium fiber supplementation (Metamucil 58.6% oral powder, 2 tablespoons every 12 hours); increased oral fluid intake; ongoing behavioral counseling |
|  | **9c** | Changes in therapeutic intervention (with rationale) | Case Presentation — no changes to therapy required; complete clinical response achieved on initial conservative regimen; no escalation to topical therapy, biofeedback, or surgical intervention |
| **Follow-up and Outcomes** | **10a** | Clinician and patient-assessed outcomes (if available) | Case Presentation — complete resolution of rectal bleeding and pain at two month follow up; no recurrence of hematochezia; patient reported full symptom resolution |
|  | **10b** | Important follow-up diagnostic and other test results | Case Presentation — clinical follow up only; no repeat endoscopy required given complete symptomatic resolution; no further diagnostic testing performed |
|  | **10c** | Intervention adherence and tolerability (How was this assessed?) | Case Presentation — assessed by patient report at two month follow up clinic visit; patient reported adherence to psyllium supplementation and behavioral modification; no adverse effects reported |
|  | **10d** | Adverse and unanticipated events | Case Presentation — none; no adverse effects from psyllium supplementation; no unexpected clinical events during follow up period |
| **Discussion** | **11a** | A scientific discussion of the strengths AND limitations associated with this case report | Discussion — strengths: comprehensive infectious exclusion including GC/chlamydia/LGV; original mechanism diagram; complete two month outcome; high-resolution endoscopic and histologic figures. Limitations: no defecography or MRI pelvis to formally document occult prolapse; single institution; single patient |
|  | **11b** | Discussion of the relevant medical literature with references | Discussion — references [1–10] including SRUS systematic review [3], mucosal prolapse syndrome [6], 116-patient series [4], sexually transmitted proctitis in HIV [8], biofeedback data [10] |
|  | **11c** | The scientific rationale for any conclusions (including assessment of possible causes) | Discussion — circumferential shear forces during occult internal mucosal prolapse as mechanistic explanation for kissing configuration; histopathologic triad as gold standard for diagnosis independent of endoscopic prolapse visibility [6,7] |
|  | **11d** | The primary take-away lessons of this case report (without references) in a one paragraph conclusion | Discussion final paragraph — SRUS presenting as kissing ulcers is an underrecognized phenotype; histopathology is essential; systematic infectious exclusion is required in HIV; conservative therapy is highly effective |
| **Patient Perspective** | **12** | The patient should share their perspective in one to two paragraphs on the treatment(s) they received | Patient perspective not included in manuscript per patient request and institutional privacy guidelines; written consent for case publication was obtained |
| **Informed Consent** | **13** | Did the patient give informed consent? Please provide if requested | YES — written informed consent was obtained from the patient for publication of this case report and all accompanying clinical images, in accordance with institutional guidelines and the Declaration of Helsinki |

*CARE guidelines: Gagnier JJ et al. The CARE guidelines: consensus-based clinical case reporting guideline development. J Med Case Rep. 2013;7:223.*
